# Comparison of PAS and adenoids in patients with and without maxillary micrognathia before orthodontic treatment

**DOI:** 10.1007/s00784-024-05657-8

**Published:** 2024-04-16

**Authors:** Maike Tabellion, Jan Lucas Felix Gustav Schneider, Constanze Charlotte Linsenmann, Jörg Alexander Lisson

**Affiliations:** Department of Orthodontics, Saarland University, Homburg, Saar Germany

**Keywords:** Maxillary micrognathia, Posterior airway space, Adenoids, Nasopharynx

## Abstract

**Objective:**

Craniofacial anomalies are widely discussed as predisposing factors of breathing disorders. Since many more cofactors exist, this study investigated the association between maxillary micrognathia and morphological changes of posterior airway space and adenoids in these patients.

**Material and methods:**

Cephalometric radiographs of *n* = 73 patients were used for data acquisition. The patients were divided into two groups according to certain skeletal characteristics: maxillary micrognathia (*n* = 34, 16 female, 18 male; mean age 10.55 ± 3.03 years; defined by a SNA angle < 79°) and maxillary eugnathia (*n* = 39, 19 female, 20 male; mean age 10.93 ± 3.26 years; defined by a SNA angle > 79°). The evaluation included established procedures for measurements of the maxilla, posterior airway space and adenoids. Statistics included Kolmogorov–Smirnov-, T- and Mann–Whitney-U-Tests for the radiographs. The level of significance was set at *p* < 0.05.

**Results:**

The cephalometric analysis showed differences in the superior posterior face height and the depth of the posterior airway space at palatal level among the two groups. The depth of the posterior airway space at mandibular level was the same for both groups, just as the size of the area taken by adenoids in the nasopharynx.

**Conclusions:**

Skeletal anomalies affect the dimension of the posterior airway space. There were differences among the subjects with maxillary micrognathia and these with a normal maxilla. However, the maxilla was only assessed in the sagittal direction, not in the transverse. This study showed that the morphology of the maxilla relates to the posterior airway space whereas the adenoids seem not to be affected.

**Clinical relevance:**

Maxillary micrognathia is significantly associated with a smaller depth of the posterior airway space at the palatal level compared to patients with maxillary eugnathia.

## Introduction

The tube-shaped pharynx consists of three parts: nasopharynx, oropharynx and hypopharynx, starting at the cranial base and ending at the sixth cervical vertebra. An open upper airway is indispensable for nasal breathing and improves growth of craniofacial structures [18]. Therefore, it is of great interest for orthodontists, pediatricians, ENT specialists and speech therapists. Respiratory function and its impact on craniofacial growth have been investigated even more controversially. Because of the close relationship between the posterior airway space and the craniofacial structures an interaction must occur. Obstruction of the upper airway is a predisposing factor for breathing problems [16]. Pharyngeal dimensions are associated with craniofacial anomalies such as maxillary or mandibular micrognathia [1]. Class II malocclusions are seen as consequence of a posterior position of the tongue impacting the cervical region and its respiratory function resulting in mouth breathing or wrong deglutition [2]. For treatment planning pharyngeal morphology is useful to be taken in consideration with the orthodontic diagnosis. Sorensen et al. [24] described the relationship of the mandible and the posterior airway space as more important than the one of the maxilla. Ceylan et al. [2] controverted this relationship.

## Aims of the study

Since many different conclusions of craniofacial anomalies and their relationship with the pharyngeal dimension coexist, this study investigated the influence of the maxillary relationship on the depth of the posterior airway space and the adenoids. Therefore, research was restricted to growing patients without any orthodontic treatment only, because the morphology of the maxilla changes due to orthodontic appliances. The use of landmarks on cephalometric radiographs should be verified as a probable method to analyze the maxillary position and dimension of the posterior airway space as well as the size of the area taken by the adenoids.

## Material and methods

### Patients

The patients were divided into two groups (maxillary micrognathia and maxillary eugnathia), and compared to each other. Cephalometric radiographs of 73 non-syndromic patients (34 maxillary micrognathia, 39 maxillary eugnathia – including 7 with maxillary prognathia) at the age of 10.55 ± 3.03 years (maxillary micrognathia) and 10.93 ± 3.26 years (maxillary eugnathia) were retrospectively identified and analyzed. All patients were exclusively diagnosed for orthodontic treatment at Saarland University Hospital.

### Inclusion/Exclusion criteria

The presence of maxillary micrognathia (SNA angle < 79°) and mandibular eugnathia (SNB angle < 81°, ANB angle < 0°) for group 1 (*n* = 34) and maxillary eugnathia or prognathia (SNA angle > 79°) and mandibular eugnathia or retrognathia (SNB angle < 81°, ANB angle > 0°) for group 2 (*n* = 39) were the inclusion criteria. The limit for SNA angle for maxillary eugnathia was set at 79° to 83°. The limit for SNB angle for mandibular eugnathia was set at 77° to 81° [6]. Exclusion criteria included mandibular macrognathia, comorbid syndromes and genetic disorders.

As a precondition, diagnostic data including digital cephalometric radiographs had to be present. Data were extracted from before the beginning of orthodontic treatment at the age of ten to thirteen or rather fourteen years.

### Control group

All patients with maxillary micrognathia (*n* = 34) were matched with patients with maxillary eugnathia (*n* = 39, including the 7 patients with maxillary prognathia). The control did not receive prior orthodontic treatment either. Patients selected for control were otherwise healthy individuals who presented themselves for treatment of non-skeletal malocclusions, e.g. crowding.

### Cephalometric measurement

A total of 73 cephalometric radiographs of patients with and without maxillary micrognathia from one orthodontic clinic were available. A subdivision by gender was not performed. The cephalometric radiographs were measured using the software OnyxCeph® 3TM (Image Instruments GmbH, Chemnitz, Germany).

### Landmarks and measuring technique

**Fig. 1:**
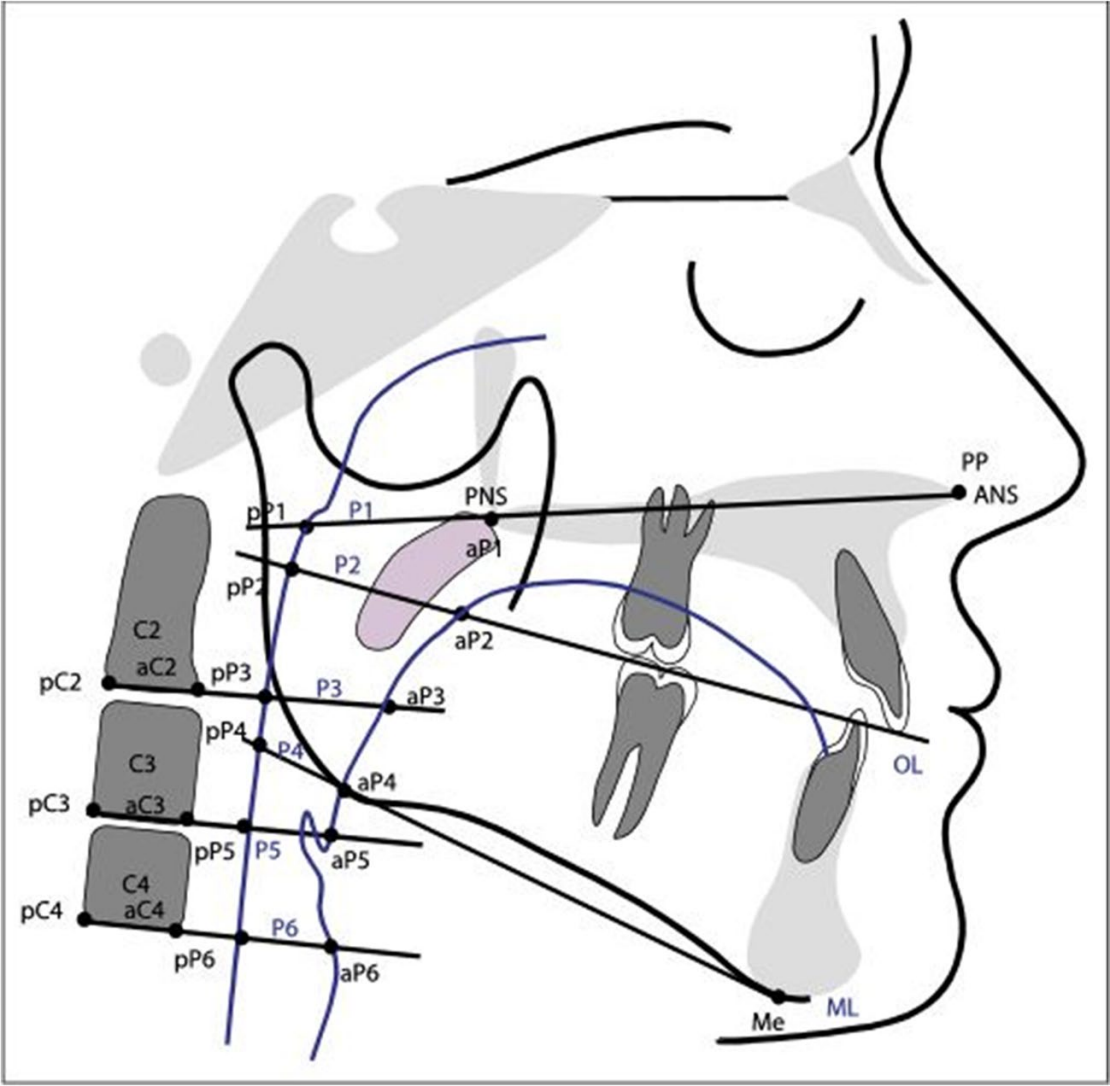
Overview of the landmarks used on the cephalometric radiographs and the linear and angular parameters calculated from them according to Kinzinger et al.

**Fig. 2:**
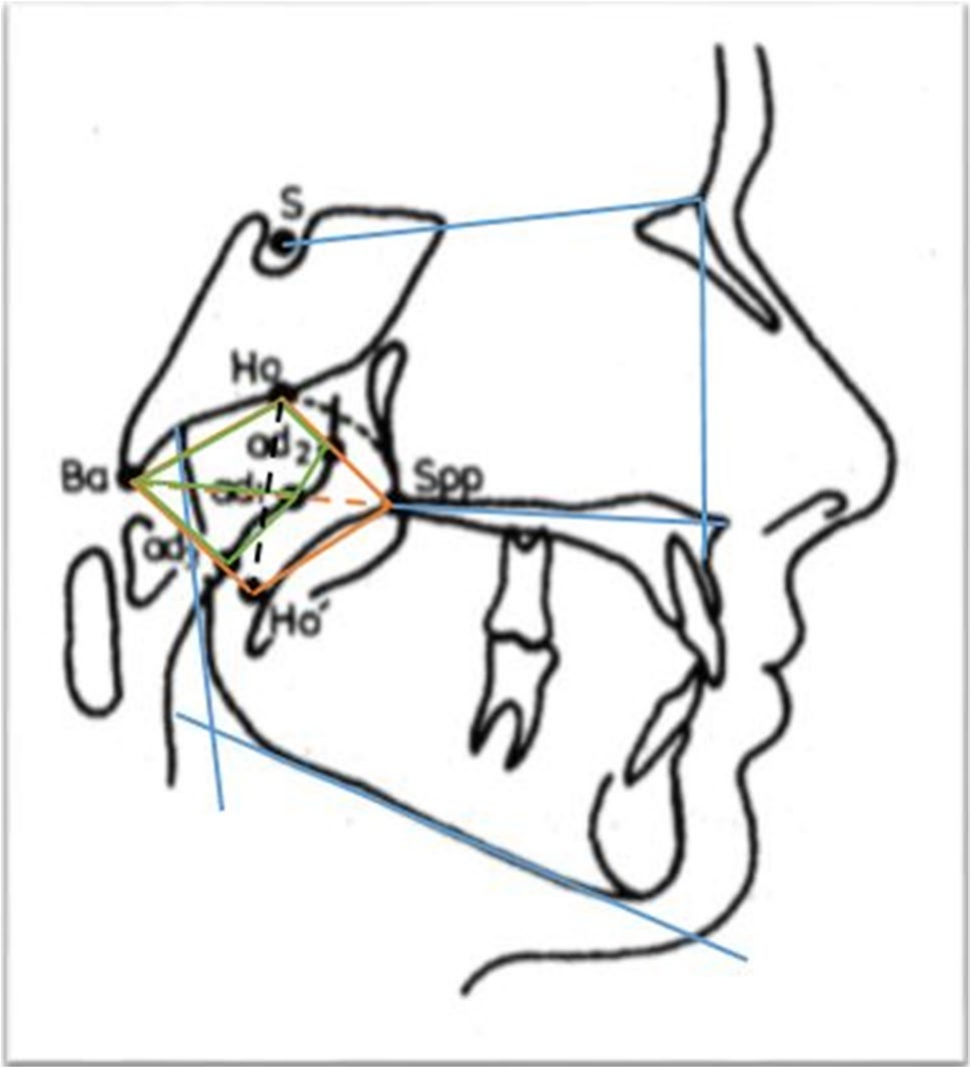
Overview of the landmarks used on the cephalometric radiographs and the linear and angular parameters calculated from them according to Jonas and Mann

The parameters for evaluation of the cephalometric radiographs were based on landmarks defined and used by Kinzinger et al. [10] and Jonas and Mann [9] for calculating distances and angles (Table 1) in all groups (Fig. 1 and 2).

**Table 1:** Cephalometric landmarks and measurements

Measurement	
Distances (mm)	
S-Ba	length of the clivus: distance between the central point of the sella turcica (Sella, (S)) and the most inferior posterior point of the anterior border of the foramen magnum (Basion, (Ba))
S-Spp	length of the posterior upper face height: distance between landmark Sella (S) and the most posterior point on the maxilla (Spina nasalis posterior, (Spp)/posterior nasal spine, (PNS))
Ba-Spp	depth of the bony nasopharynx: distance between landmark Basion (Ba) and Spina nasalis posterior (Spp)
P1	distance of the point of intersection of the nasal line and the posterior pharyngeal wall (posterior nasopharynx, (pP1)) and the point of intersection of the nasal line and the anterior pharyngeal wall (anterior nasopharynx (aP1); analog points: Spina nasalis posterior (Spp)/posterior nasal spine, (PNS))
P2	distance of the point of intersection of the occlusal plane and the posterior pharyngeal wall (superior posterior oropharynx, (pP2)) and the point of intersection of the occlusal plane and the anterior pharyngeal wall (superior anterior oropharynx, (aP2))
P3	distance of the point of intersection of the distance of the most anterior and posterior inferior point of the vertebral body C2 (aC2-pC2) and the posterior pharyngeal wall (inferior posterior oropharynx, (pP3)) and the point of intersection of the distance aC2-pC2 and the anterior pharyngeal wall (inferior anterior oropharynx, (aP3)) at the level of C2
P4	distance of the point of intersection of the mandibular line and the anterior pharyngeal wall (superior posterior laryngopharynx, (pP4)) and the point of intersection of the mandibular line and the anterior pharyngeal wall (superior anterior laryngopharynx, (aP4)) at the mandibular level
P5	distance of the point of intersection of the distance of the most anterior and posterior inferior point of the vertebral body C3 (aC3-pC3) and the posterior pharyngeal wall (inferior posterior laryngopharynx, (pP5)) and the point of intersection of the distance aC3-pC3 and the anterior pharyngeal wall (inferior anterior laryngopharynx, (aP5)) at the level of C3
P6	distance of the point of intersection of the distance of the most anterior and posterior inferior point of the vertebral body C4 (aC4-pC4) and the posterior pharyngeal wall (posterior subglottic area, (pP6)) and the point of intersection of the distance aC4-pC4 and the anterior pharyngeal wall (anterior subglottic area, (aP6)) at the level of C4
Areas (mm^2^)	
Spp-Ho-Ba-Spp	area of the bony nasopharynx: measured between the landmark Spina nasalis posterior (Spp), the most posterior intersection of the Os sphenoidale and the vomer (Hormion, (Ho)) and the landmark Basion (Ba)
Spp-Ho-Ba-Ho´-Spp	dimension of the entire nasopharynx: measured between the landmarks Spina nasalis posterior (Spp), Hormion (Ho), Basion (Ba) and the projection of Ho about the distance Ba-Spp (Hormion´, (Ho´))
ad2-Ho-Ba-ad1-ad2	dimension of the adenoids in the area of the bony nasopharynx: measured between the point of intersection of the line Ho-Spp and the posterior pharyngeal wall (ad2), the landmarks Hormion (Ho), Basion (Ba) and the point of intersection of the line Ba-Spp and the posterior pharyngeal wall (ad1)
ad2-Ho-Ba-ad3-ad2	overall dimension of the adenoids in the entire nasopharynx: measured between the landmarks ad2, Hormion (Ho), Basion (Ba) and the point of intersection of the line Ba-Ho´ and the posterior pharyngeal wall (ad3)
Percentages (%)	
ad2-Ho-Ba-ad1-ad2/Spp-Ho-Ba-Spp	adenoids in relation to the bony nasopharynx
ad2-Ho-Ba-ad3-ad2/Spp-Ho-Ba-Ho´-Spp	adenoids in relation to the entire nasopharynx
Angles (°)	
SNA	angle between the cranial base (SN) and the deepest point on the curvature of the anterior surface of the maxilla (Point A, (A))
SNB	angle between the cranial base (SN) and the deepest point on the curvature of the anterior surface of the mandibula (Point B, (B))
ML-NL	angle between the mandibular plane (ML) and the distance Spa-Spp (nasal line, (NL))
MeGoAr	gonial angle: angle between the most inferior point of the mandibular symphysis (Menton, (Me)), the most inferior posterior point of the mandibular angle (Gonion, (Go)) and the intersection of the dorsal contour of the condylar head and the contour of the posterior cranial base (Articulare, (Ar))

The angles SNA, SNB, ML-NL, MeGoAr were used to evaluate the sagittal and vertical position of maxilla and mandibula and the growth pattern.

### Statistical method, error of the method

Statistical analysis was performed with the SPSS software version 27 (IBM, Armonk, NY, USA). Statistics included Kolmogorov–Smirnov-, T- and Mann–Whitney-U-Tests for the cephalometric radiographs. The level of significance was set at *p* < 0.05. The significance level was defined as follows: *p* ≥ 0.05 not significant, *p* < 0.05 significant, *p* < 0.01 highly significant and *p* < 0.001 most highly significant. The effect size was tested by the formula r = Z/√N using Cohen´s criteria (for r): 0.1–0.3 = small effect size and low correlation, 0.3–0.5 = moderat effect size and correlation, > 0.5 = large effect size and high correlation. For testing the intrarater-reliability the evaluation process was repeated on 25 percent of each group three months after the first investigation to evaluate the impact of landmarking errors, which involved removing and replacing the markings. The differences were statistically analyzed using Dahlberg´s error of the method (MF) with the formula MF = √(∑d^2^/2n), where *d* is the difference between two measurement results and *n* is the number of duplicate measurements [3]. The MF for angular and linear measurements in the present study was < 1 for all measurements.

## Results

### Cephalometric measurements

#### Bony structures (Table 2)

**Table 2:** Clivus length and superior posterior face height [mm] for MMP and MEP

	MMP	MEP	*P* value^a^
	M ± SD	M ± SD	
Bony structures			
Clivus length	35.02 ± 5.01	36.55 ± 4.51	0.178
Superior posterior face heigth	42.19 ± 4.12	43.71 ± 2.59	0.601

Pretreatment visit, *M* Mean, *SD* standard deviation, ^a^T-test between groups

For maxillary micrognathia patients (MMP) the length of the clivus was smaller than for maxillary eugnathia patients (MEP) (MMP: 35.02 ± 5.01 mm; MEP: 36.55 ± 4.51 mm; *p* = 0.178). For MMP the posterior upper face height was smaller than for MEP (MMP: 42.19 ± 4.12 mm; MEP: 43.71 ± 2.59 mm; *p* = 0.601).

#### Posterior airway space (Table 3)

**Table 3:** Posterior airway space depth [mm] for MMP and MEP

	MMP	MEP	*P* value^a^
	M ± SD	M ± SD	
Posterior airway space			
Palatal level	21.26 ± 3.86	23.30 ± 4.17	0.034
Occlusal plane level	20.19 ± 4.66	20.09 ± 3.47	0.918
C2 level	9.71 ± 2.93	9.43 ± 3.67	0.724
Mandibular level	10.98 ± 2.98	9.70 ± 3.04	0.074
C3 level	9.77 ± 4.31	9.03 ± 4.12	0.455
C4 level	13.55 ± 5.00	11.75 ± 3.68	0.149

Pretreatment visit, *M* Mean, *SD* standard deviation, ^a^T-test between groups

For MMP the depth of the posterior airway space at the palatal level was significantly smaller than for MEP (MMP: 21.26 ± 3.86 mm; MEP: 23.30 ± 4.17 mm (*p* = 0.034; r = 0,245)). For MMP the depth of the posterior airway space at the occlusal plane level was bigger than for MEP (MMP: 20.19 ± 4.66 mm; MEP: 20.09 ± 3.47 mm; *p* = 0.918). For MMP the depth of the posterior airway space at the level of C2 was bigger than for MEP (MMP: 9.71 ± 2.93 mm; MEP: 9.43 ± 3.67 mm; *p* = 0.724). For MMP the depth of the posterior airway space at the mandibular level was bigger than for MEP (MMP: 10.98 ± 2.98 mm; MEP: 9.70 ± 3.04 mm; *p* = 0.074). For MMP the depth of the posterior airway space at the level of C3 was bigger than for MEP (MMP: 9.77 ± 4.31 mm; MEP: 9.03 ± 4.12 mm; *p* = 0.455). For MMP the depth of the posterior airway space at the level of C4 was bigger than for MEP (MMP: 13.55 ± 5.00 mm; MEP: 11.75 ± 3.68 mm; *p* = 0.149).

#### Adenoids size and percentages (Table 4)

**Table 4:** Adenoids size [mm^2^] and percentages [%] for MMP and MEP

	MMP	MEP	*P* value^a^
	M ± SD	M ± SD	
Adenoids size			
Superior area	193.49 ± 63.71	196.77 ± 43.90	0.796
Entire area	317.94 ± 103.45	335.28 ± 76.63	0.415
*Percentage*			
Adenoids/bony nasopharynx	68.12 ± 10.60	69.96 ± 11.31	0.492
Adenoids/entire nasopharynx	56.52 ± 11.67	60.06 ± 12.04	0.207

Pretreatment visit, *M* Mean, *SD* standard deviation, ^a^T-test between groups

For MMP the superior area of the adenoids was smaller than for MEP (MMP: 193.49 ± 63.71 mm^2^; MEP: 196.77 ± 43,90 mm^2^; *p* = 0.796). For MMP the entire area of the adenoids was smaller than for MEP (MMP: 317.94 ± 103.45 mm^2^; MEP: 335.28 ± 76.63 mm^2^; *p* = 0.415). For MMP the percentage of the adenoids in relation to the bony nasopharynx was smaller than for MEP (MMP: 68.12 ± 10.60%; MEP: 69.96 ± 11.31%; *p* = 0.492). For MMP the percentage of the adenoids in relation to the entire nasopharynx was smaller than for MEP (MMP: 56.52 ± 11.68%; MEP: 60.06 ± 12.04%; *p* = 0.207).

#### Angles (Table 5)

**Table 5:** Angles [°] for MMP and MEP

	MMP	MEP	*P* value^a^
	M ± SD	M ± SD	
Angles			
ML-NL	27.03 ± 5.82	25.70 ± 7.30	0.397
MeGoAr	127.23 ± 5.63	126.43 ± 6.59	0.585

Pretreatment visit, *M* Mean, *SD* standard deviation, ^a^T-test between groups

For MMP the angle ML-NL was bigger than for MEP (MMP: 27.03 ± 5.82°; MEP: 25.70 ± 7.30°; *p* = 0.397). For MMP the angle MeGoAr was bigger than for MEP (MMP: 127.23 ± 5.63°; MEP: 126.43 ± 6.59°; *p* = 0.585).

## Discussion

### Eligibility of the imaging

Adequate dimensions of the airway are prerequisites for normal breathing. Many studies showed that skeletal anomalies are predisposing factors for upper airway obstruction [8]. Cephalometric radiographs have been used widely for evaluation of craniofacial growth. Analyses for dental and skeletal anomalies and the soft tissue have been established. With cephalometric radiographs it is possible to evaluate anomalies in sagittal and vertical direction, but not in the transverse. The resulting limitation has been discussed controversially with regard to the evaluation of the posterior airway space and the adenoids. Some authors recommend other techniques for the evaluation of the upper airway, such as CT scans [7], fluoroscopy [25], fiberoptic pharyngoscopy [19] or magnetic resonance imaging [20]. In dentistry, these techniques are normally not available or indicated. Beyond that cephalometric radiographs are less expensive and carried out with less radiation. Particularly the evaluation of distances and areas are discussed as meaningful parameters to define the airway characteristics. Therefore, cephalometric radiographs are seen as valid diagnostic tools. There are many studies existing using this approach [4, 15, 22, 26]. The accuracy could have been optimized using cone beam computed tomography (CBCT) for three-dimensional analysis. The radiation exposure is less than for computed tomography, but indication setting is strict [13].

Many factors affect the upper airway such as the size of the adenoids, the length and axial area of the airway and the patient being positioned during taking the radiograph. Especially in younger patients, the size of the adenoids changes continuously and seems to be stable at the age of 14 to 15 years. Before that age, adenoids mostly affect the nasopharyngeal volume. The volume of the posterior airway space varies furthermore depending on the respiratory cycle influencing the measurements of the cephalometric radiographs. This problem can be solved by instructing patients to hold their breath during the X-ray. Therefore, cephalometric radiographs are helpful for screening, but for diagnosis of airway obstruction further otolaryngologic diagnostic techniques are required [5].

Since orthodontic appliances tend to affect the morphology of the maxilla, our research was restricted to growing patients without any orthodontic treatment only. At this age, cephalometric radiographs are useful to get an overview of the respiratory airway and the adenoids especially regarding craniofacial growth [11, 14, 23, 27]. Nevertheless, the observational data of the study must be interpreted in association with growth processes. Knowledge of the development of the nasopharyngeal zone is for interpretation mandatory [10]. For adult patients with obstructive airway problems, other otolaryngologic diagnostic tools are needed.

Patients with maxillary prognathia or mandibular retrognathia were included in the study. These sagittal anomalies of the jaws affect the posterior airway space. Nevertheless, they had to be included to the study to build a control group for comperative data generation. Otherwise, the size of the control group would have been too small for valid conclusions.

### Growth of nasopharynx and adenoids

The cephalometric radiographs used in this study have been taken during the growth spurt. During this, the tonsilla pharyngea is significantly involved in the depth of the airway. Some studies resulted in different growth pattern of the adenoids not following the growth of the rest of the lymphoid tissues. Increased growth and degeneration appear alternately with two growth peaks at the age of five and nine to twelve years [12]. Preston et al. [18] talk about the maximum of the lymphoid thickness at the age of eleven to twelve years for boys and thirteen to fourteen years for girls. Park et al. [17] proved the correlation of hypo- and hyperdivergent craniofacial growth with regard to different growth pattern and intensity of the degeneration of the tonsilla pharyngea. On these grounds measurements of the depth of the nasopharynx of patients with maxillary micrognathia make sense. Growth of the nasopharynx follows the growth of the body in a constant way. The increase in height is particularly linear and leads back to the subsidence of the hard palate. Scheerer and Lammert [21] incorporated adults in their study. They described an increasing volume of the nasopharynx because of remodeling processes of the maxilla. They also had plaster models of the nasopharynx and gave evidence of its transverse dimension. The width of the nasopharynx did not increase significantly mainly because auf increasing growth of the eustachian tube. These reports confirmed cephalometric radiographs as a useful method for evaluation of the height and depth of the airway. Despite the limitations, cephalometrcic radiographs are valid tools for evaluation of the respiratory airway, especially of the retronasal area and the adenoids.

## Conclusion

Patients with maxillary micrognathia showed a bigger extrathoracic airway than the patients with maxillary eugnathia apart from the nasopharynx. Its sagittal dimension tends to be smaller in case of maxillary micrognathia. Bony variations are a probable reason for this because the size of the lymphatic tissues of the nasopharynx was almost indistinguishable. According to the results, maxillary micrognathia was significantly associated with a smaller depth of the posterior airway space at the palatal level compared to patients with maxillary eugnathia. Since obstruction of the upper airway is a predisposing factor for breathing problems, an open upper airway is indispensable for nasal breathing and improves growth of craniofacial structures.

For a final assessment, an interference of hyperdivergent growth pattern and enlarged tonsillae palatinae as well as the different occlusions should be proved.

## Data Availability

No datasets were generated or analysed during the current study.
